# DPTEdb, an integrative database of transposable elements in dioecious plants

**DOI:** 10.1093/database/baw078

**Published:** 2016-05-12

**Authors:** Shu-Fen Li, Guo-Jun Zhang, Xue-Jin Zhang, Jin-Hong Yuan, Chuan-Liang Deng, Lian-Feng Gu, Wu-Jun Gao

**Affiliations:** ^1^College of Life Sciences, Henan Normal University, Xinxiang 453007, China; ^2^School of Basic Medical Sciences, Xinxiang Medical University, Xinxiang 453003, China; ^3^Basic Forestry and Proteomics Center, Haixia Institute of Science and Technology (HIST), Fujian Agriculture and Forestry University, Fuzhou 350002, China

## Abstract

Dioecious plants usually harbor ‘young’ sex chromosomes, providing an opportunity to study the early stages of sex chromosome evolution. Transposable elements (TEs) are mobile DNA elements frequently found in plants and are suggested to play important roles in plant sex chromosome evolution. The genomes of several dioecious plants have been sequenced, offering an opportunity to annotate and mine the TE data. However, comprehensive and unified annotation of TEs in these dioecious plants is still lacking. In this study, we constructed a dioecious plant transposable element database (DPTEdb). DPTEdb is a specific, comprehensive and unified relational database and web interface. We used a combination of *de novo*, structure-based and homology-based approaches to identify TEs from the genome assemblies of previously published data, as well as our own. The database currently integrates eight dioecious plant species and a total of 31 340 TEs along with classification information. DPTEdb provides user-friendly web interfaces to browse, search and download the TE sequences in the database. Users can also use tools, including BLAST, GetORF, HMMER, Cut sequence and JBrowse, to analyze TE data. Given the role of TEs in plant sex chromosome evolution, the database will contribute to the investigation of TEs in structural, functional and evolutionary dynamics of the genome of dioecious plants. In addition, the database will supplement the research of sex diversification and sex chromosome evolution of dioecious plants.

**Database URL**: http://genedenovoweb.ticp.net:81/DPTEdb/index.php

## Introduction

Transposable elements (TEs) are DNA elements that are capable of moving from one place in the genome to another. They contribute greatly to eukaryotic genomes, particularly plant genomes, and can account for up to 90% of the genome size in several plant species (1, 2). TEs are classified into two classes based on their mode of transposition. Class I elements are also known as retrotransposons that propagate via an RNA intermediate. These elements use a ‘copy and paste’ mechanism to insert themselves into a new location in the genome. Class II elements include DNA transposons, which move through a direct ‘cut-and-paste’ mechanism (3). The two classes can be further subdivided into orders, superfamilies and then families based on structural features. Retrotransposons are further divided into two groups, namely, long terminal repeat (LTR) elements, such as members of the Ty1/Gypsy and Ty3/Copia superfamilies, and the non-LTR elements that lack LTR sequences (e.g. long interspersed nuclear elements or LINEs, short interspersed nuclear elements or SINEs) (3). DNA transposons are further classified into three main subclasses, namely, terminal inverted repeats (TIRs), Helitrons and Mavericks (4).

Contrary to their initial portrayal as ‘junk’ DNA, increasing investigations suggested that TEs play vital roles in the genomes they occupy, such as impacting the structure and function of the host genome (5), influencing the evolutionary trajectories of their host (6), regulating gene expression of host genes (7) and donating new genes to the host genome (8). Recent studies have suggested that TEs may also be main contributors in diversification and evolution of sex chromosomes (9–11). In contrast to those of mammals and birds, the sex chromosomes of plants have evolved much more recently. Such species with ‘young’ sex chromosomes offer a unique opportunity to elucidate the mechanism of the very early stages of sex chromosome evolution (12, 13). TEs have been observed to accumulate in the sex chromosomes of a number of dioecious plant species, such as *Carica papaya* (14–16), *Silene latifolia* (17–20), *Rumex acetosa* (21), *Cannabis sativa* (22, 23) and *Bryonia dioica* (24). The identification of TEs in the genome of dioecious plants will facilitate the investigation of the influence of TEs on the structural, functional and evolutionary dynamics of the sequenced genomes, particularly on the origin and evolution of sex chromosomes.

More plant genomes, including several dioecious plants, such as *C.* *papaya* (25), *C.* *sativa* (26), *Populus trichocarpa* (27), *Morus notabilis* (28) and *Phoenix dactylifera* (29, 30), have been sequenced with the development of next-generation sequencing. In addition, the genomes of the dioecious plant *Asparagus officinalis* (31) and *S. latifolia* (32) have been sequenced and partially assembled. However, the TE annotation of these genomes is incomplete and is based on different methods. For better usage and comparison of TEs in dioecious plants, comprehensive and unified annotation of TEs in these plants is necessary. A number of databases concerning TEs are currently available. These databases can be divided into two main types. One emphasizes the analysis and classification of TEs in the context of the tree of life, such as GyDB (33) and Repbase (34). The other focuses on the identification and characterization of TEs in a specific species, such as BmTEdb (35) and MnTEdb (36). However, no databases concerning the TEs of dioecious plants exist at present.

In this study, we used a combined method to identify, classify and annotate TEs in the genomes of sequenced dioecious plants. All identified TEs were organized and deposited in a dioecious plant transposable element database (DPTEdb). Useful tools were also integrated for the analysis of TEs. We believe that DPTEdb can be used to study the origin, amplification and evolutionary dynamics of TEs in dioecious plants. DPTEdb establishes a foundation for further comparative analysis among different dioecious species to decipher the roles of TEs on plant sex chromosome evolution. 

## Construction and Content of the Database

### System implementation

The server of DPTEdb was constructed using Linux openSUSE 13.1, Apache 2.4.10, MySQL Server 5.5.33-MariaDB and Perl v5.18.1/PHP 5.4.20. All TE data and information were stored in MySQL tables for efficient management, search and display. Common Gateway Interface programs were mainly developed using Perl, JavaScript and PHP programming languages. The JBrowse Genome Browser that was built with HTML5 and JavaScript was used to manipulate and display the genome coordinates of TEs in the assemblies of eight dioecious plants in the DPTEdb (37).

### Data sources

The DPTEdb houses the information on TEs from eight dioecious plants, including *C. papaya*, *C. sativa*, *P. trichocarpa*, *M. notabilis*, *P. dactylifera*, *A. officinalis*, *Humulus scandens* and *S. latifolia*. The downloaded hyperlink for the assembly genome sequences of *C. papaya*, *C. sativa*, *P. trichocarpa*, *M. notabilis*, *P. dactylifera* and *S. latifolia* are listed in Table 1. The partial genomes of *A. officinalis* and *H. scandens* were assembled in our lab from Illumina sequencing data.

**Table 1.** List of dioecious plant species analyzed in this study

Plant species	URL	References
*C. papaya*	ftp://ftp.ncbi.nlm.nih.gov/genomes/all/GCA_000150535.1_Papaya1.0	(25)
*C. sativa*	ftp://ftp.ncbi.nlm.nih.gov/genomes/all/GCA_000230575.1_canSat3	(26)
*P. trichocarpa*	ftp://ftp.ncbi.nlm.nih.gov/genomes/all/GCA_000002775.2_Poptr2_0	(27)
*M. notabilis*	http://morus.swu.edu.cn/morusdb/	(28)
*P. dactylifera*	ftp://ftp.ncbi.nlm.nih.gov/genomes/all/GCA_000413155.1_DPV01	(29, 30)
*A. officinalis*	http://datadryad.org/resource/doi:10.5061/dryad.s71t4	(31)
*H. scandens*	Our own data	unpublished
*S. latifolia*	http://w3lamc.umbr.cas.cz/lamc/?page_id=8, respectively	(32)

### Identification of TEs in the eight dioecious plant genomes

TE libraries of the eight dioecious plants were generated using three different approaches. (1) Signature-based identification of TEs. For retrotransposons, LTR_FINDER (v1.05) (38) and MGEScan-nonLTR (v2) (39) programs were used with default parameters to search against the assemblies of the eight dioecious plant genomes to identify the LTR and non-LTR retrotransposons, respectively. HelitronScanner (40), which was developed based on the local combinational variable algorithm (41), was used to detect Helitron transposons with default parameters. For MITE transposons, MITEHunter (42) was used to search the assemblies with default parameters. (2) Similarity-based identification of TEs. The assemblies of the eight dioecious plant genomes were searched for further similarity-based identification of TEs against repeat databases. The TE sequences were detected using RepeatMasker (www.repeatmasker.org) to search against the Repbase database (34). Results with scores <250 or those with target coverage <40% were discarded. (3) *De novo* identification of TEs. *De novo* identification of TEs was performed using PILER (v1.0) (43), RepeatScout (v1.0.5) (44) and RepeatModeler (http://www.repeatmasker.org/Repeat Modeler. html, version 1.0.7). First, the assemblies of the eight dioecious plants were analyzed to identify putative TEs using the three software programs. The putative TEs that have >90% sequence similarity to each other were then discarded. Finally, the putative TEs with >90% sequence similarity to another prediction (signature-based identification and similarity-based identification) were removed to reduce the redundancy of similar predictions.

### Definition of superfamily and families of putative TEs

The putative TEs identified by the methods mentioned above were classified and annotated by comparing with Repbase using RepeatMasker, and the best hit target TE was selected as the superfamily of the analyzed TEs. Furthermore, the putative TEs were categorized into families based on the 80–80–80 rule; that is, two TE elements were classified into the same family if they shared at least 80% of sequence identity in at least 80% of their coding or internal domain, within their terminal repeat region, or in both regions on segments longer than 80 bp (3).

## Results

### Identification of TEs in eight dioecious plants

Using the methods described earlier, a total of 31 340 TEs belonging to 3446 TE families were identified in the eight dioecious plant genomes. These TEs and families were organized into an easy-to-use web-based database, DPTEdb; the composition of which is presented in Table 2. A total of 2458, 5622, 11 673, 7071 and 4038 TEs were detected from the entire genome sequences of *C. papaya*, *C. sativa*, *P. trichocarpa*, *M. notabilis* and *P. dactylifera*, respectively. Apparently, retrotransposons were more abundant than DNA transposons in these five species (Table 1). For the three plants with assemblies representing a minority of the genome, only 408, 35 and 35 TEs were identified from *A. officinalis*, *H. scandens* and *S. latifolia*, respectively. In the future, more TEs will be identified when the comprehensive genome information for these species is available.

**Table 2.** Summary of identified TEs in eight dioecious plant assemblies

			*C. papaya*	*C. sativa*	*P. trichocarpa*	*M. notabilis*	*P. dactylifera*	*A. officinalis*	*H. scandens*	*S. latifolia*
Class	Order	Superfamily	Members/families	Members/families	Members/families	Members/families	Members/families	Members/families	Members/families	Members/families
Retrotransposons	LTR	*Caulimovirus*		1/1	6/5	3/1	24/2			
		*Copia*	184/43	941/36	1557/60	1832/48	882/75	3/3		
		*DIRS*	2/2	5/5	5/5	9/8	14/9			
		*ERV1*	6/5	184/13	52/18	35/17	70/36			
		*ERV4*				1/1	8/3			
		*ERVK*	2/1	7/7	10/8	263/8	27/17			
		*ERVL*			2/2	4/1	3/3			
		*Gypsy*	617/20	1230/31	5587/39	2182/50	1001/64	1/1		
		*Ngaro*		1/1	6/4	6/5	6/3			
		*Pao*	1/1	72/8	140/8	121/6	24/13			
		Unknown	1110/42	1110/202		1514/92	605/234			7/2
	LINE	*CR1*						1/1		
		*CRE*								1/1
		*DRE*		2/2						
		*I*				2/2	1/1	2/2		
		*Jockey*		1/1						
		*L1*	69/49	992/111	87/19	19/19	145/17	58/52	12/12	5/5
		*L2*								1/1
		*Penelope*		1/1			1/1			
		*R2*				1/1				
		*Rex*						1/1		
		*RTE*				29/1				
		*RTE-BovB*					4/4	4/4		1/1
		*Tad1*					1/1			
Subtotal			1991/163	4547/419	7452/168	6021/260	2816/483	70/64	12/12	15/10
DNA tranposons	TIR	*Academ*					1/1			
		*CMC*	2/2	20/17	5/2	2/2	7/7	13/13		8/8
		*hAT*	1/1	34/27	17/14	6/5	19/17	26/26	9/9	1/1
		*MULE*	1/1	73/66	5/5	5/5	22/14	34/33		3/3
		*Novosib*								1/1
		*p*				1/1	1/1			
		*PIF-Harginger*	3/2	26/26	3/3	7/7	47/43	7/7	1/1	
		*TcMar*	1/1	3/3	3/2	1/1	31/30	1/1	8/8	2/1
		*Unknown*			2770/136			2/2		
	MITE	*MITE*	2/2	122/70	78/41	150/60	103/65	76/26		
	Helitron	*Helitron*	457/152	797/158	1340/177	878/77	991/299	179/175	5/5	5/4
Subtotal			467/161	1075/367	4221/380	1050/158	1222/477	338/283	23/23	20/18
Total			2458/324	5622/786	11 673/548	7071/418	4038/960	408/347	35/35	35/28

### Web interface and usage

**Figure 1.**
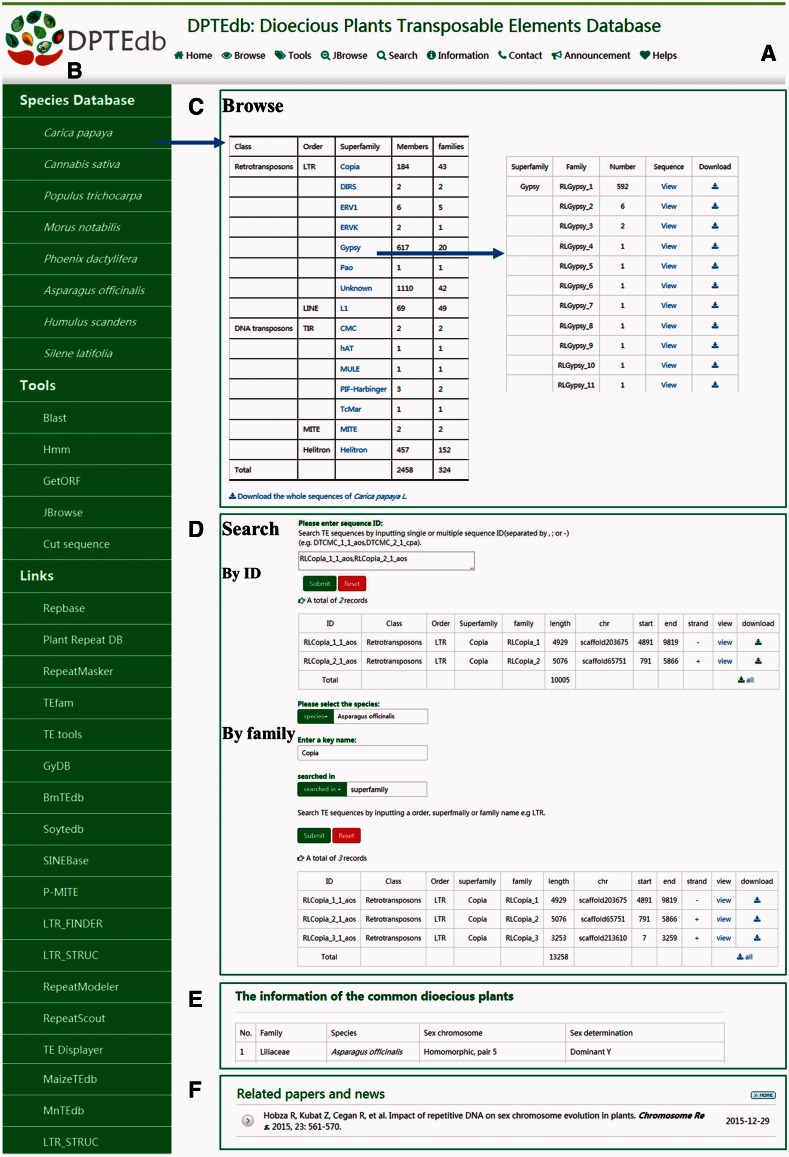
Interface and some functional sections of DPTEdb. **(A)** Top menu of DPTEdb. **(B)** Side menu of DPTEdb. **(C)** Browsing interface of database DPTEdb. **(D)** Searching interface of DPTEdb. **(E)** A table embedded in the DPTEdb, containing the information of the commonly studied dioecious plants. **(F)** Related publications and news.

DPTEdb is an integrated dioecious plant TE database that provides a comprehensive platform to study TEs in dioecious plants, as well as materials for further studies on the genome evolution of dioecious plants. An efficient and user-friendly web-based user interface was built based on different classifications of TEs from eight dioecious plants. Users could easily browse and search for the TE information and perform various analyses using the analysis tools. Data download and export are also available. A top menu (Figure 1A) and a side menu (Figure 1B) were designed to navigate the DPTEdb content. The top menu includes four main sections, namely, Browse, Tools, Search and Information. The side menu contains three sections, namely, Species Database, Tools and Links. In the homepage, we also provide a summary of this database and display news and publications related to dioecious plants (Figure 1F).

### Browse

Users can browse in the top menu or species database in the side menu to browse and visualize the basic information of the TEs in a selected plant species through hyperlinks of the species name. For each species, the TEs identified were grouped into different superfamilies, which can be browsed using the hyperlinks provided in the website (Figure 1C). In the ‘TE superfamily statistical information’ page, clicking the corresponding entry of each family allows users to obtain the detailed information of this family, including classification, length, location and nucleotide sequences.

### Search and download

In the search interface of DPTEdb, we provide two pathways for users, namely, sequence ID(s) and search keyword(s). Users can use a specific sequence ID to search the DPTEdb and find corresponding entries. When using a keyword to search the DPTEdb, users should select one species first and enter a keyword afterward (TE order, superfamily or family name) to locate the information of interest quickly. After searching, a new webpage will open to display all the matched results, which can be printed out as a tabular format output or downloaded by clicking the corresponding hyperlinks (Figure 1D). Users can download all TE sequences or TE sequences of interest by order, superfamily or family in a given species.

### Tools

**Figure 2.**
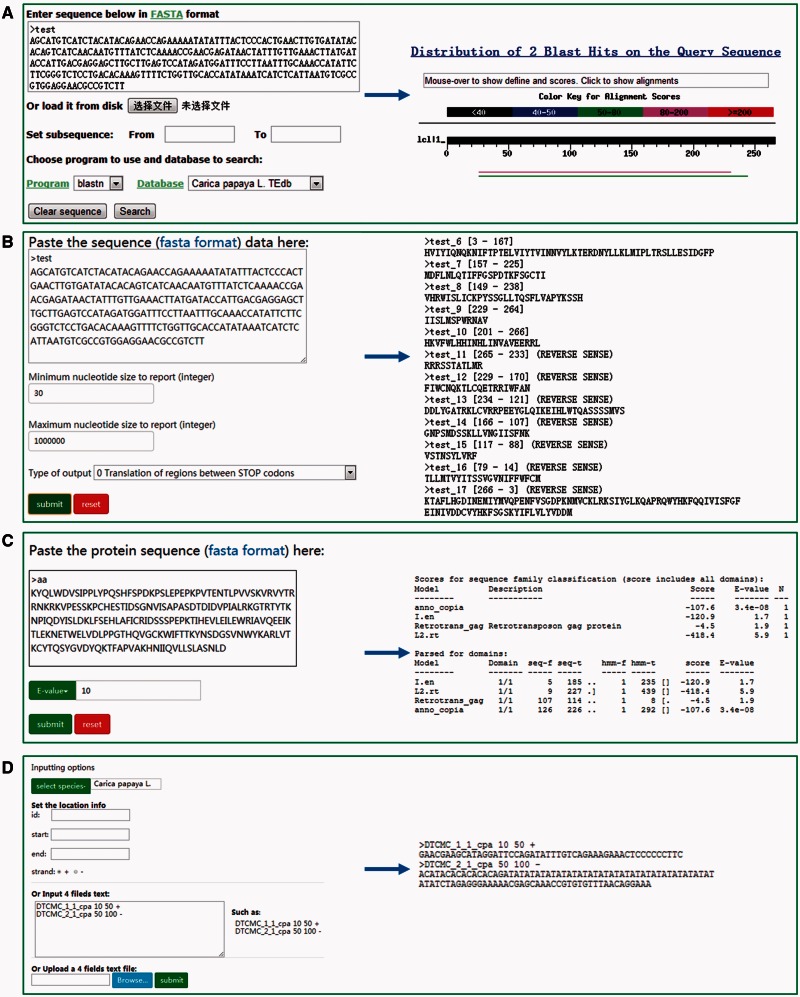
Different analysis tools provided in DPTEdb. **(A)** BLAST interface of DPTEdb. A sample of BLASTn results is shown. **(B)** GetORF interface of a test DNA sequence and snapshots of output results. **(C)** Interface of HMMER and a sample of protein sequence analyzed by HMMER. **(D)** Snapshots of the interface of the Cut sequence tool.

**Figure 3.**
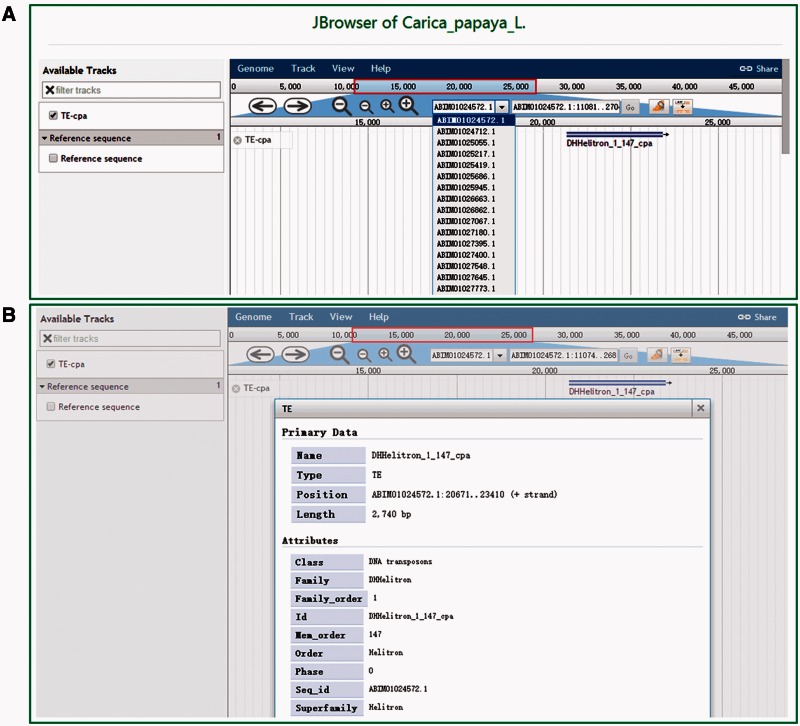
Snapshots of the Jbrowse tool of DPTEdb. **(A)** Genome sequence view of a region in scaffold ABIM01024572.1 from *C. papaya*. **(B)** Detailed information of the TE DHHelitron_1_147_cpa in the graphic interface.

DPTEdb offers five sequence analysis tools (BLAST, GetORF, HMMER, Gbrower and Cut sequence) to facilitate users to analyze the TE data. Users can submit any query sequences to do BLASTN or tBLASTN against the DPTEdb for homology search (Figure 2A). Users can also analyze the potential open reading frame (ORF) of a query sequences using the GetORF tool (Figure 2B). HMMER is provided to facilitate the identification and classification of TEs. In this page, Hidden Markov model (HMM) profile of coding domains of retrotransposons were collected from previous studies. Users can use the ORFs obtained by GetORF to search against these TE HMM models using HMMER package for the identification and classification of the query sequences (Figure 2C). Furthermore, users can obtain a sequence or sequences in a defined position using the Cut sequence tool (Figure 2D). The genome browser based on Jbrowse was also used to display the coordinates of TEs in the genomes of the eight dioecious plants in the DPTEdb. Users can click the Jbrowse tool in the top menu and select one species to browse on a large scale in a graph visualization interface (Figure 3A). Users can also conveniently view the detailed information of TEs by simply clicking the name of the TE in the graphic interface (Figure 3B). The information on reference sequences and TEs could be selectively shown on Jbrowse by clicking ‘Select Tracks’ button to set output items.

### Information and links

In the information section, we provided a table that shows the sex chromosome and sex determination information of 65 commonly studied dioecious plants (Figure 1E). The information could help users understand these dioecious plants quickly. In the side menu, a number of links to other database and software websites relevant to DPTEdb were provided. Users can reach these websites easily by clicking the corresponding links (Figure 1B).

## Discussion

An increasing number of studies have indicated that TEs may play important roles in the diversification and evolution of sex chromosomes (12, 45, 46). Identification of TEs in the genome of dioecious plants could establish a foundation for study of the roles of TEs played on the genome of the sequenced dioecious plant. Comparison of TEs and other repetitive sequences among different dioecious plants will facilitate the investigation of the contributions of TEs to the sex diversification and sex chromosome evolution (21, 47). We constructed a database called DPTEdb for better usage and comparative analysis of TEs in dioecious plants. DPTEdb is manually curated and dedicated to TE identification and classification in the genomes of dioecious plants using comprehensive and unified annotation approaches, serving as the initial TE data repository for dioecious plants. DPTEdb provides interactive access to the database content via a series of user interfaces and batch/bulk download opportunities. Several analysis tools were also impeded in the DPTEdb for convenient and efficient analysis of TEs, such as homology comparison using BLAST and classification of TEs using GetORF and HMMER. Furthermore, DPTEdb offers information on commonly studied dioecious plants, as well as news and publications related to dioecious plants. This can help users understand dioecious plants conveniently.

Submissions of new TE data to DPTEdb for dioecious plants are invited and highly encouraged. Among the eight included dioecious plants, a large number of TEs were identified and classified in the five species with draft genomes. In contrast, only a small number of TEs were detected in *A. officinalis*, *H. scandens* and *S. latifolia*, in which the assemblies only represent a minority of their entire genome. With increasing genome sequences of dioecious plants, we will improve and continuously update the DPTEdb for the TEs of the existing species, other dioecious plant species, and closely related hermaphroditic taxa.

## Conclusion

We have generated a comprehensive TE database for dioecious plants called DPTEdb. The current DPTEdb includes 31 340 TEs from eight dioecious plants, along with classification information. DPTEdb allows users to search and browse TEs within the eight dioecious plants and permits batch download and analysis of TE sequences using the interfaces and analysis tools. Therefore, this database can contribute to the study of TEs in dioecious plants, and establishes a foundation for further comparative and evolutionary studies of sex chromosomes in plants.

## Accessibility

The DPTEdb database is freely available at http://genedenovoweb.ticp.net:81/DPTEdb/index.php.

## Funding

This work was supported by grants from the National Natural Science foundation of China (31300202, 31470334) and a grant from Key Technologies R&D Program of Henan province (132102110195). Funding for open access charge: National Natural Science Foundation of China.

*Conflict of interest*. None declared.
